# Effects of Sodium Octanoate on Innate Immune Response of Mammary Epithelial Cells during *Staphylococcus aureus* Internalization

**DOI:** 10.1155/2013/927643

**Published:** 2012-12-24

**Authors:** Nayeli Alva-Murillo, Alejandra Ochoa-Zarzosa, Joel E. López-Meza

**Affiliations:** Centro Multidisciplinario de Estudios en Biotecnología, Facultad de Medicina Veterinaria y Zootecnia, Universidad Michoacana de San Nicolás de Hidalgo, Km 9.5 Carretera Morelia-Zinapécuaro, Posta Veterinaria, 58893 Morelia, MICH, Mexico

## Abstract

Bovine mammary epithelial cells (bMECs) are capable of initiating an innate immune response to invading bacteria. Short chain fatty acids can reduce *Staphylococcus aureus* internalization into bMEC, but it has not been evaluated if octanoic acid (sodium octanoate, NaO), a medium chain fatty acid (MCFA), has similar effects. In this study we determined the effect of NaO on *S. aureus* internalization into bMEC and on the modulation of innate immune elements. NaO (0.25–2 mM) did not affect *S. aureus* growth and bMEC viability, but it differentially modulated bacterial internalization into bMEC, which was induced at 0.25–0.5 mM (*~*60%) but inhibited at 1-2 mM (*~*40%). Also, bMEC showed a basal expression of all the innate immune genes evaluated, which were induced by *S. aureus*. NaO induced BNBD4, LAP, and BNBD10 mRNA expression, but BNBD5 and TNF-**α** were inhibited. Additionally, the pretreatment of bMEC with NaO inhibited the mRNA expression induction generated by bacteria which coincides with the increase in internalization; only TAP and BNDB10 showed an increase in their expression; it coincides with the greatest effect on the reduction of bacterial internalization. In conclusion, NaO exerts a dual effect on *S. aureus* internalization in bMEC and modulates elements of innate immune response.

## 1. Introduction

The epithelium is an important line of defense against pathogenic microorganisms. In cattle, bovine mammary epithelial cells (bMECs) are responsible for the production of milk but contribute significantly to the immune defense of the mammary gland [1]. bMECs are able to generate a variety of inflammatory mediators such as cytokines, chemokines, and antimicrobial peptides (APs) in response to invading pathogens, which indicates that these cells are capable of initiating an *in vivo* innate immune response to pathogenic bacteria [2, 3]. Additionally, stimulation of bMEC with bacteria (*Escherichia coli *or* Staphylococcus aureus*) or their components induces a strong innate immune response [4, 5].

Mammary glands of lactating cows are frequently infected by pathogens causing bovine mastitis, which is a major disease affecting the dairy industry resulting in economic losses and decreased animal health [6]. This pathology is mainly caused by *S. aureus* that has the ability to internalize into bMEC and survive within them, which leads to a low response to conventional antibiotic therapy and to the establishment of subclinical and chronic mastitis [7, 8]. Thus, alternative methods are required to control bovine mastitis. In this sense, an alternative that has received great attention in the last years comprises the modulation of innate immune response of the mammary gland to facilitate the elimination of invading pathogens [1].

*S. aureus* internalization into bMEC is considered an important pathogenic mechanism for the establishment of mastitis. In previous studies, we have shown that short chain fatty acids (SCFAs) propionic, butyric, and hexanoic, some of them components of bovine milk, inhibit *S. aureus* internalization into bMEC and regulate the expression of innate immunity response genes [9, 10]. In the same way, we demonstrated that other components of bovine milk, as vitamin D (cholecalciferol), also reduce *S. aureus* internalization and differentially regulate AP expression in bMEC [11]. These studies demonstrate that SCFAs and vitamin D could be used as effective innate immunity modulators through their induction or addition in mammary gland, which might lead to a better defense against bacterial infection.

Octanoic acid (caprylic acid) is a medium chain fatty acid (MCFA) component of human and bovine milk, which inactivates human pathogens as viruses and bacteria [12, 13]. Furthermore, Nair et al. [14] showed that caprylic acid and its monoglyceride (monocaprylin) inactivate common mastitis pathogens, including *S. aureus*. However, the role of this MCFA on *S. aureus* internalization into bMEC remains unknown. In this work, we assess the role of NaO in internalization of *S. aureus* responsible of mastitis into bMEC. Also, we evaluated the gene expression of elements of innate immune response during this process.

## 2. Materials and Methods 

### 2.1. Strain and Reagents

*Staphylococcus aureus* subsp. *aureus *(ATCC 27543) strain isolated from a case of bovine clinical mastitis was used in this study. This strain has recognized capacity to internalize into bovine mammary epithelial cells [18]. The bacterial inoculum was obtained from cultures grown overnight at 37°C in Luria-Bertani broth (LB, Bioxon, México) and CFUs were adjusted by measuring optical density at 600 nm. Sodium octanoate was purchased from Sigma (St. Louis, MO, USA) and the working solutions were dissolved in water. Based on previous studies with SCFAs on *S. aureus* invasion in bMEC, we established a range of concentrations of 0.25 to 2 mM to carry out the experiments [9, 10].

### 2.2. Primary Culture of Bovine Mammary Epithelial Cells (bMEC)

bMECs were obtained from alveolar tissue from udders of lactating cows as described [19]. Cells from passages 2nd to 8th were cultured in Petri dishes (Corning-Costar, NW, USA) in growth medium (GM) composed by DMEM medium/nutrient mixture F-12 Ham (DMEM/F-12 K, Sigma) supplemented with 10% fetal calf serum (Equitech-Bio Inc, Kerrville, TX, USA), 10 *μ*g/mL insulin (Sigma), 5 *μ*g/mL hydrocortisone (Sigma), 100 U/mL penicillin and streptomycin (100 *μ*g/mL), and 1 *μ*g/mL amphotericin B (Invitrogen, Carlsbad, CA, USA). Cells were grown in 5% CO_2_ atmosphere at 37°C. Only bMECs with 80–90% confluence were utilized in this study.

### 2.3. Effect of Sodium Octanoate on *S. aureus* 27543 Growth and bMEC Viability

To analyze NaO effect on *S. aureus *growth, 9 × 10^7^ CFUs/mL were cultured at 37°C in LB broth supplemented with different concentrations of this molecule (0.25–2 mM) and growth was monitored turbidimetrically (600 nm) after 24 h. To determine the effect of NaO on bMEC viability, 5 × 10^3^ cells were incubated with 0.25–2 mM NaO during 24–48 h at 37°C in a 96-well flat-bottom plate. Then, 10 *μ*L of 5 mg/mL of 3-(4,5-dimethyl-2-thiazolyl)-2,5-diphenyl-2H-tetrazolium bromide (MTT, Sigma) solution in phosphate buffer saline (PBS) was added to each well and incubated during 4 h at 37°C. Finally, 100 *μ*L of acid isopropanol (95% isopropanol and 5% of 1 N HCl) was added to dissolve formazan crystals. Optical density was measured with a microplate spectrophotometer (DAS) at 595 nm and compared with untreated controls.

### 2.4. Effect of Sodium Octanoate on Internalization of *S. aureus* 27543 into bMEC

We used bMEC-polarized monolayers that were created on plates coated with 6–10 *μ*g/cm^2^ rat-tail type I collagen (Sigma). Internalization experiments were carried out as described using gentamicin protection assay [9]. Briefly, prior to assays bMEC were incubated with different NaO concentrations (0.25–2 mM) for 24 h in F12 K medium without antibiotics and serum. bMEC monolayers (~2 × 10^5^ cells/well) were challenged with *S. aureus* (30 : 1 bacteria per cell), for this, bMECs were inoculated with 65 *μ*L of bacterial suspensions to 9.2 × 10^7^ CFU/mL and incubated for 2 h in 5% CO_2_ at 37°C. After, bMEC monolayers were washed three times with PBS (pH 7.4) and incubated in F12 K medium without serum and antibiotics, supplemented with 50 *μ*g/mL gentamicin for 1 h at 37°C to eliminate extracellular bacteria. Then, bMEC monolayers were detached with trypsin-EDTA (Sigma) and lysed with 250 *μ*L of sterile distilled water. bMEC lysates were diluted 100-fold, plated on LB agar for triplicate, and incubated overnight at 37°C. The number of total CFU was determined by the standard colony counting technique. Data are presented as the percentage of internalization in relation to untreated bMEC.

### 2.5. RNA Isolation and Gene Expression Analysis

bMEC total RNA (5 *μ*g) was extracted from all conditions evaluated with Trizol reagent (Invitrogen) according to manufacturer's instructions and then used to synthesize cDNA. Genomic DNA contamination was removed from RNA samples with DNase I treatment (Invitrogen). cDNA synthesis was carried out as previously described [10].

The relative quantification of gene expression (qPCR) was performed using the comparative Ct method (ΔΔCt) in a StepOne Plus Real-Time PCR System (Applied Biosystems) according to manufacturer's instructions. The reactions were carried out with a SYBR Green PCR Master Mix (Applied Biosystems, Carlsbad, CA, USA). Specific primers were used to amplify genes encoding AP and TNF-*α* (Table 1). GAPDH was used as an internal control [20].

### 2.6. Data Analysis

Data were obtained from three independent experiments performed by triplicate and compared by Student's *t*-test, except qPCR data analysis, in this case an analysis of variance (ANOVA) was carried out. The results are reported as mean ± the standard errors (SE). *P* values of <0.05 were considered significant.

## 3. Results

### 3.1. Effect of Sodium Octanoate on *S. aureus* Growth and bMEC Viability

**Figure 1:**
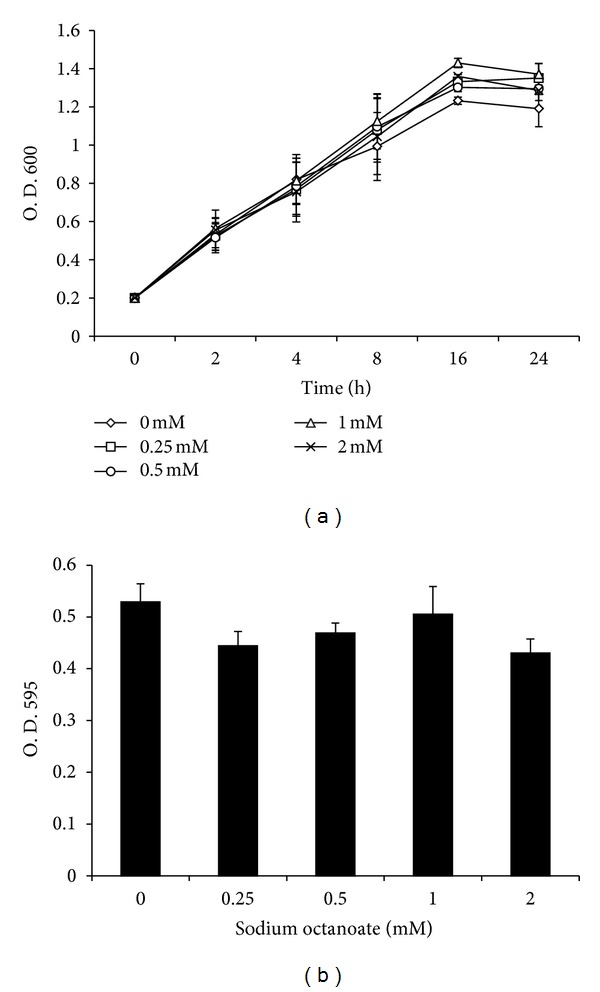
Effect of sodium octanoate on *S. aureus* growth and bovine mammary epithelial cell viability. (a) *S. aureus* was cultured in the presence of NaO and bacteria growth was monitored turbidimetrically (600 nm) after 24 h. (b) bMECs were cultured with NaO and viability was determined by MTT assay at 24 h. In all cases, concentrations evaluated were 0.25, 0.5, 1 and 2 mM. Each point in (a) or each bar in (b) shows the mean of triplicates ± SE of three independent experiments.

Caprylic acid has antimicrobial activity against mastitis pathogens [14]. Thus, we evaluated the effects of NaO (0.25 to 2 mM) on *S. aureus* growth. The results showed that NaO did not affect bacterial growth after 24 h (Figure 1(a)). These results establish that NaO has no antimicrobial effect under the conditions evaluated.

The effect of NaO on viability of bMEC was evaluated by MTT assay. The results showed that this MCFA has no effect on bMEC viability, since for all conditions the values of viability obtained were higher than 96% (Figure 1(b)). Thus, in the conditions and times evaluated, NaO was not toxic to bMEC.

### 3.2. Sodium Octanoate Differentially Modulates the Internalization of *S. aureus* into Bovine Mammary Epithelial Cells

**Figure 2:**
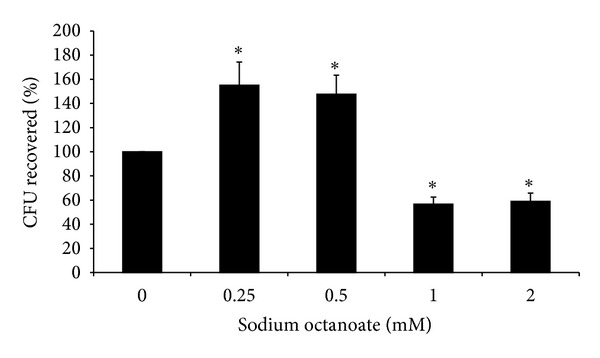
Sodium octanoate differentially modulates *S. aureus* internalization into bMEC. Effect of NaO on *S. aureus* internalization into bMEC is represented by the percentage of CFU recovered after bMEC lysis. Values were determined considering the control (without MCFA) as 100% internalization. Each bar shows the mean of triplicates ± SE of three independent experiments. The symbol “∗” indicates significant changes (*P* < 0.05) in relation to control cells cultured without MCFA.

To determine the effect of NaO on the internalization of *S. aureus* into bMEC, we carried out gentamicin protection assays. bMECs were pretreated with different concentrations of this MCFA (0.25 to 2 mM) 24 h before the challenge with *S. aureus*. Of note, we observed that NaO has a differential effect on *S. aureus* internalization into bMEC, which was concentration dependent. According to CFU recovered, NaO at 0.25 and 0.5 mM stimulates *S. aureus* internalization into bMEC (*∼*60%) in relation to control cells (without MCFA) (Figure 2). However, bMEC treated with NaO at 1 and 2 mM showed a significant reduction in *S. aureus* internalization (*∼*40%) (Figure 2). Also, we evaluated the effect of 0.0625, 0.125, 0.375, 0.75, 1.5, and 3.5 mM NaO on *S. aureus* internalization into bMEC. NaO concentrations less than 0.5 mM stimulated *S. aureus* internalization; the highest effect was detected at 0.0625 mM (*∼*80%) (data not shown). On the other hand, concentrations higher than 0.75 mM showed an inhibitory effect on *S. aureus* internalization, starting with *∼*40% at 0.75 mM and reaching maximal effect at 3.5 mM (*∼*80%). These results indicate that NaO differentially modulates *S. aureus* internalization into bMEC in a concentration-dependent manner.

### 3.3. Effects of Sodium Octanoate on Innate Immune Elements Expression in bMEC

**Figure 3:**
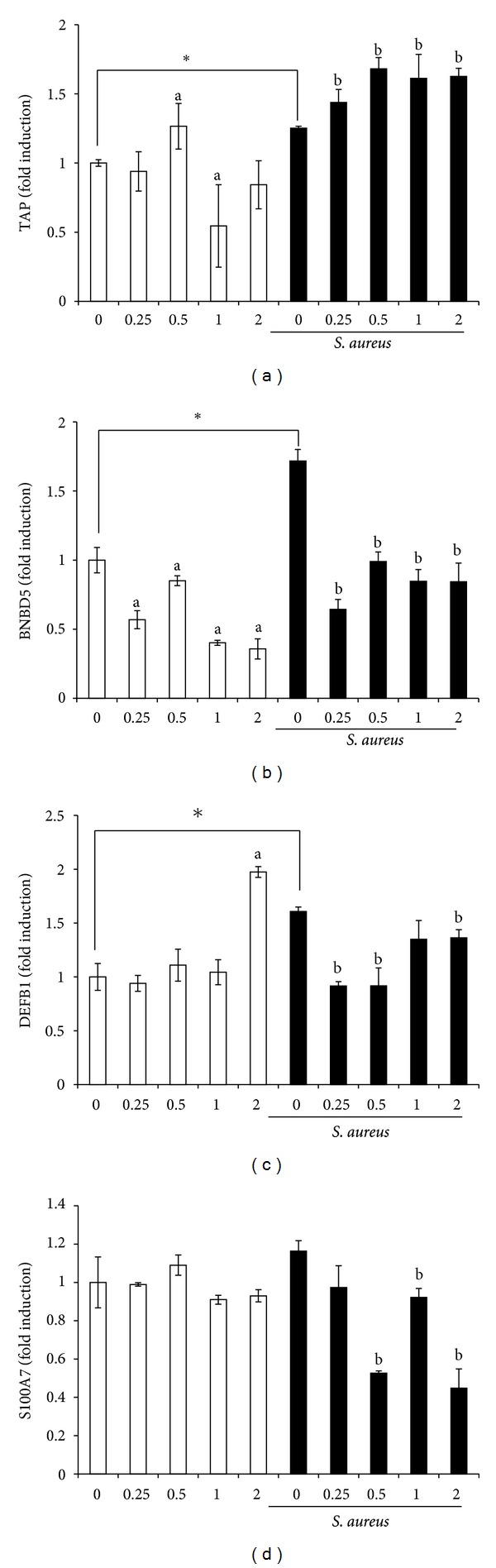
Effects of sodium octanoate on the expression of antimicrobial peptide mRNA. qPCR analysis that shows the effect on TAP (a), BNBD5 (b), DEFB1 (c), and S100A7 (d) mRNA expression. bMECs were treated with the NaO concentrations (mM) indicated (24 h) and then were challenged with *S. aureus* during 2 h. Each bar shows the mean of triplicates ± SE of three independent experiments. GAPDH was used as endogenous gene in all conditions. The symbol “∗” indicates significant changes (*P* < 0.05) in relation to unchallenged bMEC within each treatment. The letter “a” indicates significant changes (*P* < 0.05) compared to control cells without treatment. The letter “b” indicates significant changes (*P* < 0.05) compared to challenged bMEC.

**Figure 4:**
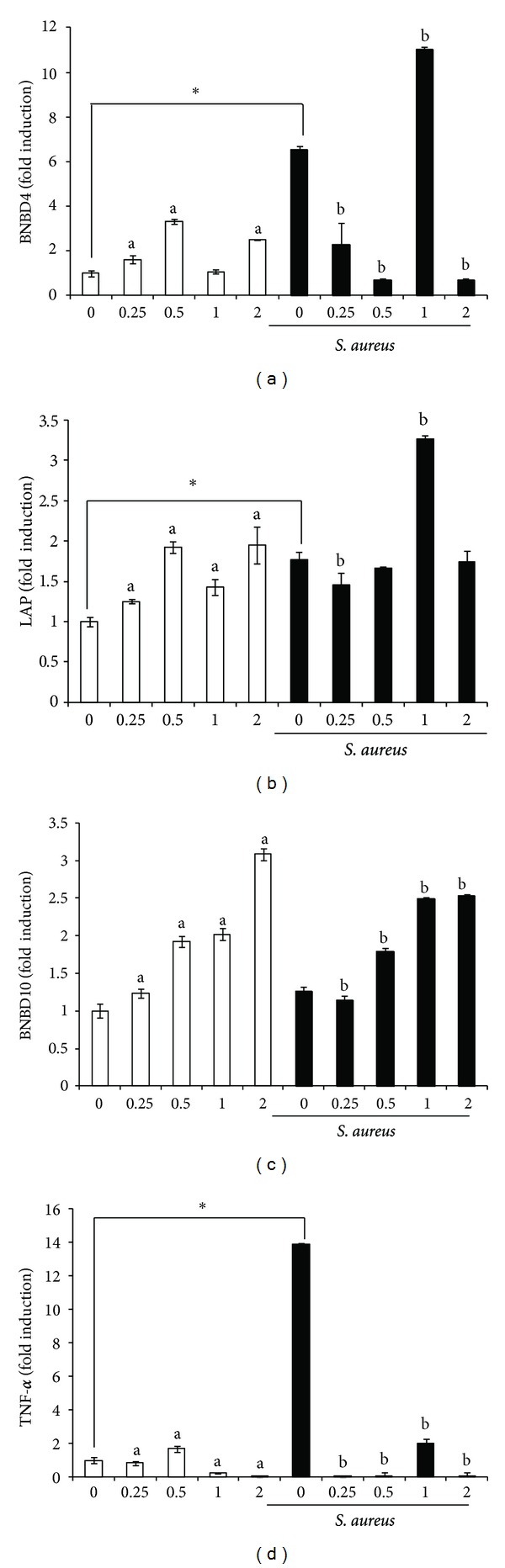
Effects of sodium octanoate on the expression of innate immune elements of bMEC. qPCR analysis that shows the effect on BNBD4 (a), LAP (b), BNBD10 (c), and TNF-*α* (d) mRNA expression. bMEC were treated with the NaO concentrations (mM) indicated (24 h) and then were challenged with *S. aureus* during 2 h. Each bar shows the mean of triplicates ± SE of three independent experiments. GAPDH was used as endogenous gene in all conditions. The symbol “∗” indicates significant changes (*P* < 0.05) in relation to unchallenged bMEC within each treatment. The letter “a” indicates significant changes (*P* < 0.05) compared to control cells without treatment. The letter “b” indicates significant changes (*P* < 0.05) compared to challenged bMEC.

The effects of NaO on transcription of innate immune genes in bMEC were analyzed by evaluating the expression of several APs and a proinflammatory cytokine. The APs tested were TAP, LAP, DEFB1, BNBD4, BNBD5, and BNBD10. Also, we evaluated S100A7, a calcium-binding protein with antibacterial activity and the cytokine TNF-*α*. The bMECs have a basal expression of all the APs genes tested (Figures 3 and 4) and NaO modulated their expression. TAP mRNA expression showed a differential expression, at 0.5 mM NaO its expression was slightly induced but at 1 mM was inhibited, the other conditions showed no changes (Figure 3(a)). Regarding to BNBD5, mRNA expression it was inhibited in all conditions (*∼*50%) (Figure 3(b)). bMEC treated with NaO did not essentially modify the expression of DEFB1 and S100A7, only at 2 mM the DEFB1 expression was increased (*∼*2-fold) (Figures 3(c) and 3(d)). Also, in almost all conditions NaO induces BNBD4, LAP and BNBD10 mRNA expression (Figure 4). Interestingly, LAP and BNBD10 mRNA expression increased in a concentration-dependent manner. 

Conversely, in Figure 4(d), we showed that NaO has a dual effect on TNF-*α* mRNA expression. NaO (0.5 mM) slightly induced the TNF-*α* mRNA expression, but with NaO 1-2 mM it was significantly inhibited. 

### 3.4. *S. aureus* Induces the Expression of Innate Immune Elements in bMEC but the Pretreatment with Sodium Octanoate Differentially Modulates this Effect

The expression of all AP and TNF-*α* genes tested were induced when bMECs were challenged with *S. aureus*, except for BNBD10 and S100A7 (Figures 3 and 4). The levels of induction were different among the genes tested; the bMEC showed only a slight upregulation for TAP while the maximal induction was observed for BNDB4 (*∼*6-fold) and TNF-*α* (*∼*14-fold). Of note, we detected that the pretreatment of bMEC with NaO inhibited the induction generated by bacteria for DEFB1, BNBD5, and TNF-*α* genes. A similar behavior was shown for BNBD4 mRNA expression, except at 1 mM, in this case we observed an increase of *∼*2-fold in relation to bMEC only infected (Figure 4(a)). On the other hand, LAP mRNA expression induced by bacteria was not modified by the pretreatment with NaO, except at 1 mM, in this condition mRNA expression was induced (*∼*2-fold) (Figure 4(b)). Finally, only TAP and BNDB10 mRNA expression showed an increase when bMECs were pretreated with NaO and then infected. This induction was concentration dependent. Also, we observed a correlation in AP mRNA expression in bMEC pretreated with NaO with respect to bacterial internalization. In the conditions where the internalization was stimulated (0.25 and 0.5 mM), the AP mRNA expression was essentially inhibited. However, the highest effect on the reduction of bacterial internalization coincides with the increase in mRNA expression for TAP, BNBD10, LAP (1 mM), and BNBD4 (1 mM).

## 4. Discussion

The present study showed that NaO differentially modulates *S. aureus* internalization into bMEC in a concentration-dependent manner. Also, NaO modulates mRNA expression of innate immune response genes. These findings suggest that NaO could have a biological significance as a regulator of innate immune defense of epithelium, in particular from mammary gland, to facilitate the elimination of invading pathogens.

Worldwide, mastitis is the most prevalent disease in dairy cattle and is caused mainly by *S. aureus*. This bacterium can internalize and survive within bovine mammary epithelial cells [7, 21, 22]. In previous studies we demonstrated that SCFAs, in particular propionic, butyric, and hexanoic (some of the components of bovine milk fat), reduce *S. aureus* internalization into bMEC and regulate AP gene expression [9, 10]. Also, we showed that other milk components, as vitamin D, could reduce *S. aureus* internalization into bMEC and modulate AP gene expression [11].

It has been established that caprylic acid and its derivatives have antimicrobial activity against enteropathogenic *Escherichia coli *[23]. In addition, Nair et al. [14] report that concentrations higher than 50 mM caprylic acid and its monoglyceride have antimicrobial activities against mastitis pathogens. According to these data, *S. aureus* growth was not affected in the presence of NaO (0.25 to 2 mM) (Figure 1(a)), probably due that we used lower concentrations than those reported with antimicrobial activity against mastitis pathogens. On the other hand, MCFAs (octanoate and decanoate) can affect cell viability and induce apoptosis [24]. For this reason, we evaluated the effects of NaO on bMEC viability. Our results showed that this MCFA did not affect bMEC proliferation (Figure 1(b)). In agreement, Harvey et al. [25] showed that MCFAs (C8:0–C12:0) did not significantly affect the growth of a primary cell line derived from human aortic endothelial cells.

Previous results of our laboratory demonstrate that SCFAs can modulate *S. aureus* internalization into bMEC [9, 10]; this led us to hypothesize that MCFAs could also regulate this process. To test this, bMECs were pretreated with NaO by 24 h and then infected. NaO (0.75, 1, 2, and 3.5 mM) decreased *S. aureus* internalization into bMEC in a concentration-dependent manner, reaching maximal inhibition of 80% (Figure 2). Recently, Kollanoor-Johny et al. [26] showed that supplementation of feed with caprylic acid reduces ~80% *Salmonella* Enteritidis invasion in chickens. Also, they established that the reduction in the pathogen ability to invade intestinal epithelial cells was related to a downregulation of the invasion genes *hilA* and *hilD *of *Salmonella*. On the other hand, we detected that lower concentrations (0.0625 to 0.5 mM) of NaO stimulated *S. aureus* internalization. In similar way, Van Immerseel et al. [27] reported that sodium acetate might increase the expression of virulence genes in *Salmonella* Typhimurium, which may facilitate bacterial invasion. In addition, caprylic acid induces PPAR*γ* mRNA expression in bovine mammary epithelial cells, which leads to the inhibition of NF-*κ*B transcription factor that plays a key role in regulating the immune response to infection [28, 29]. Also, this MCFA induces CD36 mRNA expression, whose activation in mouse is associated with Toll-like receptor 2 (TLR2) and mediates the phagocytosis of *S. aureus* [30]. To our knowledge this is the first report that shows a role of NaO on *S. aureus* internalization in bMEC. However, further studies are needed to determine the participation of NaO on the regulation of virulence genes in *S. aureus* and bMEC gene expression to establish its relevance in bacteria internalization.

The bovine mammary epithelium is able to express elements of innate immunity when mastitis pathogens invade this tissue [2, 3]. Also, bMECs are capable of initiating an *in vitro* innate immune response to invading bacteria [9, 10]. To test whether the modulation of *S. aureus* internalization into bMEC by NaO correlates with the innate immune response gene expression, we evaluated the mRNA expression of different antimicrobial molecules, six AP (*β*-defensin) genes and the antimicrobial protein S100A7.

AP expression has been reported in bovine mammary gland as well as in *in vitro* cultures of bMEC, which can be induced by bacteria or milk components [9–11, 31]. In agreement, bMEC showed a basal expression of all AP genes tested. In addition, all of them were induced by *S. aureus* (Figures 3 and 4). In general, NaO differentially modulated AP mRNA expression. In this sense, it is well known that AP expression is regulated in a tissue-specific manner at transcriptional, posttranscriptional, and posttranslational level and is stimulus-dependent [32]. We showed that NaO induced mRNA expression of BNBD4, BNBD10, and LAP in all conditions; in addition, TAP and DEFB1 mRNA expression was induced by this MCFA in some treatments (Figures 3 and 4). We do not know the mechanism by which NaO induces the expression of these APs. However, in a study carried out in rat small intestine it was shown that fatty acids of milk, including caprylic acid, enhance the expression of the CBP/p300 genes (a transcriptional coactivator), which is one of the chromatin remodeling factors and regulate histone acetylation [33]. This could explain the AP induction, but further studies are necessary to clarify the role of histone acetylation in AP expression modulated by NaO in bMEC.

On the other hand, BNBD5 and TNF-*α* mRNA expression was essentially inhibited in all conditions (Figures 3(b) and 4(d)). It has been established that BNBD5 and TNF-*α* expression in bMEC requires the participation of NF-*κ*B transcription factor [34, 35]. In a study using Caco-2 human epithelial cell line, it was shown that caprylic acid does not modify the activation state of NF-*κ*B, but inhibits the gene transcription of the chemokine IL-8, which contains NF-*κ*B-binding motifs in its promoter regions [36]. This indicates that other mechanisms of regulation that have not been described may be regulating the expression of these genes in bMEC treated with NaO. In addition, the TNF-*α* protein secretion was not modified when bMECs were treated with NaO (data not shown).

Accordingly, APs are expressed in mammary cells in response to bacterial infection [31, 37–40]. This was corroborated in this study (Figures 3 and 4). Also, NaO pretreated bMEC and then challenged with *S. aureus* showed an increase in TAP and BNBD10 expression in all conditions. In addition, LAP and BNBD4 expression was induced in some treatments. These results correlate with the reduction in *S. aureus* internalization into bMEC by NaO (Figure 2). These data are similar to that previously reported for SCFAs [9, 10]. However, further experiments are necessary in order to determine if the expression of AP is responsible for reducing *S. aureus* internalization into bMEC. On the other hand, *S. aureus* was not able to induce AP gene expression in NaO pretreated bMEC, for most of the conditions. We believe that this inhibition is due to that NaO is modifying the activation state of several unidentified transcription factors important for the expression induced by bacteria. Finally, the presence of NaO in bovine milk, together to its antimicrobial and immune effects here reported, suggest a possible role of this MCFA in modulating immune response of bovine mammary gland during mastitis. 

## 5. Conclusion

We analyzed the NaO participation during *S. aureus* internalization in a primary culture of bovine mammary epithelial cells. Our data indicate that NaO differentially modulates *S. aureus* internalization and regulates elements of innate immune response. These results point towards this MCFA being an effective modulator of innate immunity genes in mammary gland, which may lead to a better defense against bacterial infection and highlights the relevant function that epithelium plays during defense.

## Figures and Tables

**Table 1:** Primers used in this study.

Specificity		Primer sequence (5′-3′)	Fragment size (bp)	Annealing temperature (°C)	References
Tracheal antimicrobial peptide (TAP)	F R	GCGCTCCTCTTCCTGGTCCTG GCACGTTCTGACTGGGCATTGA	216	57	[10]
Bovine neutrophil *β*-defensin 5 (BNBD5)	F R	GCCAGCATGAGGCTCCATC TTGCCAGGGCACGAGATCG	143	55	[15]
Lingual antimicrobial peptide (LAP)	F R	GCCAGCATGAGGCTCCATC CTCCTGCAGCATTTTACTTGGG	194	54	[15]
Bovine neutrophil *β*-defensin 10 (BNBD10)	F R	GCTCCATCACCTGCTCCTC AGGTGCCAATCTGTCTCATGC	152	54	[11]
Bovine neutrophil *β*-defensin 4 (BNBD4)	F R	GCCAGCATGAGGCTCCATC CGTTTAAATTTTAGACGGTGT	278	54	[15]
Bovine *β*-defensin 1 (DEFB1)	F R	CCATCACCTGCTCCTCACA ACCTCCACCTGCAGCATT	185	54	[11]
Bovine psoriasin (S100A7)	F R	GCAGCTCTCAGCTTGAGCAG CCAGCAAGGACAGGAACTCAG	221	54	[11]
Tumor necrosis factor-alpha (TNF-*α*)	F R	CCCCTGGAGATAACCTCCCA CAGACGGGAGACAGGAGAGC	101	55.5	[16]
Glyceraldehyde-3-phosphate dehydrogenase (GAPDH)	F R	TCAACGGGAAGCTCACTGG CCCCAGCATCGAAGGTAGA	237	56.9	[17]
